# Intratumoral or Subcutaneous MK-2118, a Noncyclic Dinucleotide STING Agonist, with or without Pembrolizumab, for Advanced or Metastatic Solid Tumors or Lymphomas

**DOI:** 10.1158/1078-0432.CCR-24-2824

**Published:** 2025-01-23

**Authors:** Jason J. Luke, Randy F. Sweis, J. Randolph Hecht, Reva Schneider, Mark N. Stein, Talia Golan, Timothy A. Yap, Anuradha Khilnani, Mo Huang, Runchen Zhao, Thomas Jemielita, Sandip Pravin Patel

**Affiliations:** 1 UPMC Hillman Cancer Center, University of Pittsburgh, Pittsburgh, Pennsylvania.; 2 University of Chicago, Chicago, Illinois.; 3 UCLA Jonsson Comprehensive Cancer Center, Santa Monica, California.; 4 Mary Crowley Cancer Research, Dallas, Texas.; 5 Columbia University Medical Center, New York, New York.; 6 Sheba Medical Center, Derech Sheba 2 Oncology Institute, Ramat-Gan, Israel.; 7 University of Texas MD Anderson Cancer Center, Houston, Texas.; 8 Merck & Co., Inc., Rahway, New Jersey.; 9 University of California San Diego Moores Cancer Center, La Jolla, California.

## Abstract

**Purpose:**

We evaluated the noncyclic dinucleotide stimulator of IFN genes agonist MK-2118 ± pembrolizumab in participants with advanced solid tumors or lymphomas.

**Patients and Methods:**

This first-in-human study (NCT03249792) enrolled participants with refractory, advanced solid tumors or lymphomas. Participants received intratumoral (IT) MK-2118 100 to 20,000 μg (arm 1), IT MK-2118 900 to 15,000 μg plus intravenous (IV) pembrolizumab 200 mg every 3 weeks (arm 2), or subcutaneous (SC) MK-2118 5,000 to 150,000 μg plus IV pembrolizumab 200 mg every 3 weeks (arm 4); arm 3 (visceral injection of MK-2118) was not pursued. IT dosing used an accelerated titration design and modified toxicity probability interval method; SC dosing (arm 4) was started subsequent to arms 1 and 2. The primary objectives were safety/tolerability. MK-2118 pharmacokinetics was a secondary endpoint; objective responses and biomarkers were exploratory endpoints.

**Results:**

A total of 140 participants were enrolled (arm 1, *n* = 27; arm 2, *n* = 57; arm 4, *n* = 56). Grade 3/4 treatment-related adverse events occurred in 22%, 23%, and 11% of participants, respectively, but no maximum tolerated dose was identified up to MK-2118 20,000, 15,000, and 150,000 μg across the three arms. Dose-dependent increases in MK-2118 systemic exposure were observed following IT and subcutaneous administration. Objective responses were seen in 0%, 6%, and 4% of participants, respectively. IT MK-2118 led to dose-dependent changes in stimulator of interferon genes–based blood RNA expression levels, IFNγ, IFNγ-induced protein 10, and IL6; SC MK-2118 did not generate dose-related immune responses.

**Conclusions:**

IT MK-2118 ± pembrolizumab and SC MK-2118 plus pembrolizumab had manageable toxicity and limited antitumor activity. IT but not SC administration demonstrated systemic immune effects.

**Translational Relevance**

Immune checkpoint inhibitors only benefit a subset of patients as the majority have primary or secondary resistance, often due to a lack of adaptive immune priming. The cyclic guanosine monophosphate–adenosine monophosphate synthase/stimulator of interferon genes (STING) pathway has been characterized preclinically as having major potential to drive type I IFN signaling, adaptive immunity, and anticancer response. We present the phase I characterization of a novel noncyclic dinucleotide STING agonist MK-2118, by intratumoral (IT) or subcutaneous (SC) administration, with and without pembrolizumab. In this group of participants with refractory cancers, we observed major differences in IT versus SC treatment in driving both the local and systemic pharmacology of STING agonism. Although both IT and SC dosing demonstrated increases in IFNγ-induced protein 10 and IFNγ, only IT treatment demonstrated increases in peripheral blood type I IFN–associated gene expression. These data provide insight into pharmacologic and immune impacts surrounding the route of administration and can inform the further development of programs targeting STING moving forward.

## Introduction

The cyclic guanosine monophosphate–adenosine monophosphate synthase/stimulator of interferon genes (STING) pathway is a key cytosolic DNA–sensing pathway that detects nucleic acids. Aberrant cytosolic DNA in cancer leads to antigen-presenting cell activation and subsequent T-cell priming against tumor-associated antigens (1–3). Activation of the cyclic guanosine monophosphate–adenosine monophosphate synthase/STING pathway in the tumor microenvironment leads to strong induction of type I IFNs and proinflammatory cytokines (e.g., IL6, TNFα; ref. 4). This ultimately potentiates T-cell activation and enhances the ability of the innate immune system to present tumor-associated antigens to CD8^+^ T cells through antigen cross-presentation (3, 4).

Synthetic cyclic dinucleotides were developed and investigated as STING agonists with the intent of increasing potency (1). Intratumoral (IT) injection of these agents in preclinical models elicited antitumor immune responses and tumor regression in both injected and noninjected lesions (4). However, optimal dosing is not yet understood, given the nonlinear relationship between dose and tumor-specific T-cell responses, with higher doses resulting in the paradoxical killing of T cells (5). Enzymatic degradation of cyclic dinucleotides in circulating blood, particularly by ectonucleotide pyrophosphatase/phosphodiesterase 1 (6), restricts their administration to IT injection.

The limitations associated with cyclic dinucleotide STING agonists and IT administration have shifted the focus of recent research on developing more stable STING agonists that could be delivered systemically. Syngeneic mouse tumor models investigating the noncyclic dinucleotide small-molecule STING agonist MK-2118 demonstrated that IT, subcutaneous (SC), and oral administration resulted in dose-dependent antitumor activity, with complete tumor regression observed in 80% to 100% of treated animals (7). Dosing via all three routes was associated with substantial elevations of type I IFNs and proinflammatory cytokines in tumors and plasma. Importantly, in models that were partially responsive or nonresponsive to anti–programmed cell death protein 1 (PD-1) therapy, combination therapy with SC or oral MK-2118 plus anti–PD-1 therapy resulted in greater inhibition of tumor growth and prolongation of survival compared with either agent alone (7). These findings supported clinical development of the drug, and herein, we describe results from the first-in-human study (NCT03249792) of MK-2118 alone and combined with pembrolizumab.

## Patients and Methods

The study was conducted in compliance with all local and/or national regulations (including all applicable data protection laws and regulations), International Council on Harmonisation Good Clinical Practice guidelines, and the ethical principles that have their origin in the Declaration of Helsinki. The study protocol was approved by the Institutional Review Board or independent ethics committee at each study site. All participants provided written informed consent before participating in the study.

### Participants

Inclusion criteria were adults (≥18 years old) with histologically or cytologically confirmed advanced or metastatic (stages III–IV) solid tumors or lymphomas that were surgically unresectable and had been treated with, or were intolerant to, all treatments with known clinical benefit. All participants had measurable disease by Response Evaluation Criteria in Solid Tumors (RECIST) version 1.1 (solid tumors) or revised International Working Group criteria (lymphomas), an Eastern Cooperative Oncology Group performance status of 0 or 1, and adequate organ function. In addition, participants in arms 1 and 2 had to have ≥1 injectable lesion that was amenable to injection and biopsy and ≥1 discrete and/or distant noninjected lesion amenable to biopsy. Participants in arm 4 had to have ≥1 discrete lesion amenable to ≥3 separate biopsies.

### Study design and treatment

This was a phase I, multicenter, open-label study. Participants were treated with IT MK-2118 at an initial dose of 100 μg into cutaneous or SC lesions in arm 1, intravenous (IV) pembrolizumab (RRID:AB_3076193) 200 mg every 3 weeks plus IT MK-2118 at an initial dose of 100 μg into cutaneous or SC lesions in arm 2, and IV pembrolizumab 200 mg every 3 weeks plus SC MK-2118 at an initial dose of 5,000 μg in arm 4. Participants in arm 3 were to be treated with IV pembrolizumab plus IT MK-2118 into visceral lesions, but no enrollment occurred in this arm. Participants in arm 1 with progressive disease (PD) were permitted to cross over to arm 2 upon completion of the dose-limiting toxicity (DLT) evaluation period (i.e., 21 days), initiating arm 2 at screening. IT MK-2118 administration was conducted via visual inspection, palpation, or ultrasound guidance.

Arms 1 and 2 used an accelerated titration design followed by a dose escalation and confirmation IT dosing phase using the modified toxicity probability interval method. To ensure safety, the initiation of treatment arms was staggered. In arm 2, IT MK-2118 was initiated ≥2 dose levels behind the cleared MK-2118 monotherapy dose in arm 1 and could not exceed the maximum tolerated dose determined for MK-2118 monotherapy. Arm 4 was initiated once the IT MK-2118 5,000-μg dose level in arm 1 had completed DLT evaluation per the modified toxicity probability interval design.

### Endpoints and assessments

Primary endpoints were DLTs, adverse events (AEs), and discontinuation of study treatment because of AEs. The DLT evaluation period was 21 days for arms 1 and 2 and 35 days for arm 4, with AEs captured according to NCI Common Terminology Criteria for Adverse Events version 4.0 (RRID:SCR_010296).

Secondary endpoints included pharmacokinetic parameters of MK-2118. Blood samples for pharmacokinetic assessment were taken before MK-2118 dosing, at the end of MK-2118 dosing, and at 0.5, 1, 2, 4, 6, 8, 12, and 24 hours after MK-2118 dosing on day 1 of cycle 1.

Exploratory endpoints included the objective response rate (ORR), progression-free survival (PFS), overall survival (OS), and pharmacodynamic biomarkers. Images were evaluated using RECIST version 1.1 (solid tumors) or Cheson criteria (lymphomas; ref. 8); the Modified Severity-Weighted Assessment Tool was used in participants with cutaneous T-cell lymphoma (CTCL).

Peripheral blood samples for assessment of STING-based RNA expression levels were obtained before MK-2118 dosing on day 1 and between 6 and 8 hours after MK-2118 dosing on day 1 of cycle 1. The RNA signature assessed genes previously shown to be associated with the STING pathway. Blood samples for assessment of IFNγ, IFNγ-induced protein 10 (IP-10), IL6, and C-reactive protein (CRP) were obtained before MK-2118 dosing on day 1 and 2, 6, 12, and 24 hours after MK-2118 dosing on day 1 of cycle 1.

### Statistical analysis

Safety was evaluated in all participants who received ≥1 dose of treatment. The DLT-evaluable population included all participants in the safety population who were observed for safety for 21 days (arms 1 and 2) or 35 days (arm 4) after the first dose of assigned treatment or who had experienced a DLT. Rates of DLTs were estimated using the pooled adjacent violators algorithm, and 80% confidence intervals (CIs) were based on the Bayesian posterior credible intervals. Pharmacokinetic analyses were conducted in all participants who were compliant with study procedures and had available data from ≥1 study treatment. Efficacy was evaluated in all participants with a baseline imaging assessment who received ≥1 dose of study treatment (full analysis set population); responses in participants with CTCL were evaluated as a separate subgroup. For response rates, the exact method for binomial data was used to calculate 95% CIs; relationships between the best percentage change from baseline in target tumor size and programmed cell death ligand 1 (PD-L1) tumor proportion score and combined positive score were evaluated using Spearman correlations. PFS and OS were evaluated using the Kaplan–Meier method for censored data.

### Data availability

Merck Sharp & Dohme LLC, a subsidiary of Merck & Co., Inc., Rahway, NJ, USA (MSD), is committed to providing qualified scientific researchers access to anonymized data and clinical study reports from the company’s clinical trials for the purpose of conducting legitimate scientific research. MSD is also obligated to protect the rights and privacy of trial participants and, as such, has a procedure in place for evaluating and fulfilling requests for sharing company clinical trial data with qualified external scientific researchers. The MSD data-sharing website (available at https://externaldatasharing-msd.com/) outlines the process and requirements for submitting a data request. Applications will be promptly assessed for completeness and policy compliance. Feasible requests will be reviewed by a committee of MSD subject matter experts to assess the scientific validity of the request and the qualifications of the requestors. In line with data privacy legislation, submitters of approved requests must enter into a standard data-sharing agreement with MSD before data access is granted. Data will be made available for request after product approval in the USA and EU or after product development is discontinued. There are circumstances that may prevent MSD from sharing requested data, including country- or region-specific regulations. If the request is declined, it will be communicated to the investigator. Access to genetic or exploratory biomarker data requires a detailed, hypothesis-driven statistical analysis plan that is collaboratively developed by the requestor and MSD subject matter experts; after approval of the statistical analysis plan and execution of a data-sharing agreement, MSD will either perform the proposed analyses and share the results with the requestor or will construct biomarker covariates and add them to a file with clinical data that is uploaded to an analysis portal so that the requestor can perform the proposed analyses.

## Results

### Participants

A total of 140 participants (arm 1, *n* = 27; arm 2, *n* = 57; arm 4, *n* = 56) were enrolled at seven study sites in the United States and one in Israel (Supplementary Fig. S1); in arm 2, two participants were enrolled but not treated and nine participants crossed over from arm 1 for a total of 64 participants. Demographic and baseline characteristics are shown in Table 1. Overall, the median age was 59.0 years, and most participants were male (57%) and White (78%). The majority of participants (71%) had an Eastern Cooperative Oncology Group performance status of 1. The most common tumor types were colorectal cancer in 15 participants (11%), breast cancer in 13 participants (9%), and melanoma in 12 participants (9%). The study population was generally representative of the real-world setting in locations where participants were enrolled (Supplementary Table S1). Sixty percent of participants had received ≥2 prior lines of therapy. The median (range) number of administrations was 7 (1–17) doses of MK-2118 over a median (range) of 43 (1–224) days in arm 1, 7 (1–39) doses of MK-2118 plus 3 (1–35) doses of pembrolizumab over 50 (1–805) days in arm 2, and 8 (1–43) doses of MK-2118 plus 3 (1–35) doses of pembrolizumab over 57 (1–745) days in arm 4.

**Table 1.** Demographics and baseline characteristics (safety population).

	Arm 1: IT MK-2118 Monotherapy	Arm 2: IT MK-2118 + Pembrolizumab	Arm 4: SC MK-2118 + Pembrolizumab
	(*n* = 27)	(*n* = 64^a^)	(*n* = 56)
Median age (range), years	56.0 (41.0–79.0)	61.5 (19.0–89.0)	58.5 (23.0–90.0)
Age category			
<65 years/≥65 years	18 (67)/9 (33)	40 (63)/24 (38)	38 (68)/18 (32)
Sex			
Male/female	15 (56)/12 (44)	30 (47)/34 (53)	37 (66)/19 (34)
Race			
White	25 (93)	50 (78)	41 (73)
Black/African American	2 (7)	5 (8)	8 (14)
Asian	0	7 (11)	4 (7)
American Indian or Alaska Native	0	0	1 (2)
Multiple	0	1 (2)	2 (4)
Missing	0	1 (2)	0
ECOG PS			
0/1/2	10 (37)/17 (63)/0	14 (22)/50 (78)/0	19 (34)/36 (64)/1 (2)
Median TMB (range), mut/Mb^b^	4.7 (3.9–5.5)	7.0 (0–56.7)	4.7 (0–11.7)
Median lymphocyte count (range), 10^9^/L^c^	1.16 (0.50–1.83)	1.20 (0.36–2.90)	0.98 (0.08–3.10)
Median albumin levels (range), g/L^d^	40 (28–47)	40 (24–51)	41 (31–49)
Prior receipt of anti–PD-(L)1 agents	14 (52)	34 (53)	20 (36)
Number of prior lines of therapy			
0	3 (11)	12 (19)	7 (13)
1	5 (19)	11 (17)	16 (29)
≥2	15 (56)	25 (39)	16 (29)
Other	4 (15)	13 (20)	14 (25)
Missing	0	3 (5)	3 (5)

Data are *n* (%) of participants unless otherwise stated. The safety population included all participants who received ≥1 dose of study treatment.

Abbreviations: PS, performance status; TMB, tumor mutational burden.

a Nine participants in arm 1 crossed over to arm 2; these nine participants are included in both columns.

b *n* = 2 for arm 1, *n* = 33 for arm 2, and *n* = 26 for arm 4.

c *n* = 26 for arm 1, *n* = 63 for arm 2, and *n* = 54 for arm 4.

d *n* = 27 for arm 1, *n* = 64 for arm 2, and *n* = 54 for arm 4.

### Safety

#### IT MK-2118 with or without IV pembrolizumab (arms 1 and 2)

In arm 1 [*n* = 27, DLT-evaluable population (i.e., all participants who received ≥1 dose of treatment and were followed for ≥21 days or who had experienced a DLT)], a total of three DLTs (grade 3 hypotension, injection site pain, and injection site reaction) were reported with IT MK-2118 monotherapy, without an obvious dose relationship (Table 2). All 27 participants treated with IT MK-2118 monotherapy in the safety population experienced AEs (Table 3). The most common AEs (>30% of participants) were injection site pain (52%), pyrexia (41%), anemia (33%), and fatigue (33%). Treatment-related AEs were reported in 21 participants (78%) and were grade 3 or 4 in six participants (22%); there were no grade 5 treatment-related AEs. The most common treatment-related AEs (≥25% of participants) were injection site pain (52%), pyrexia (33%), and chills (26%).

**Table 2.** Summary of DLTs (DLT-evaluable population)^a^.

Treatment group	*n* (%); 80% CI	PAVA estimate (%)
Arm 1		
IT MK-2118 10,000 μg (*n* = 6)		
Total	1 (17); 4–39	10.0
Grade 3 hypotension	1 (17)	
IT MK-2118 20,000 μg (*n* = 6)		
Total	2 (33); 15–57	33.3
Grade 3 injection site pain	1 (17)	
Grade 3 injection site reaction	1 (17)	
Arm 2^b^		
IT MK-2118 10,000 μg + pembrolizumab (*n* = 17)		
Total	2 (12); 4–24	9.7
Grade 3 cytokine release syndrome	1 (6)	
Grade 3 injection site reaction	1 (6)	
IT MK-2118 15,000 μg + pembrolizumab (*n* = 14)		
Total	1 (7); 1–19	9.7
Grade 3 injection site reaction	1 (7)	
Arm 4		
SC MK-2118 20,000 μg + pembrolizumab (*n* = 7)		
Total	1 (14); 3–35	3.6
Grade 3 pneumonitis	1 (14)	

Abbreviation: PAVA, pooled adjacent violators algorithm.

a Only the dose groups in which DLTs were reported are shown. No other DLTs occurred. The DLT-evaluable population included all participants who received ≥1 dose of treatment and were followed for ≥21 days (arms 1 and 2) or ≥35 days (arm 4) or who had experienced a DLT.

b Includes all DLTs experienced while receiving IT MK-2118 + pembrolizumab in the 55 participants originally assigned to arm 2 and in the nine participants in arm 1 after crossing over to arm 2.

**Table 3.** Most common AEs and all immune-mediated AEs (safety population).

	Arm 1: IT MK-2118 Monotherapy	Arm 2: IT MK-2118 + Pembrolizumab	Arm 4: SC MK-2118 + Pembrolizumab
	(*n* = 27)	(*n* = 64^a^)	(*n* = 56)
Participants with ≥1 AE	27 (100)	62 (97)	56 (100)
Most common AEs^b^			
Pyrexia	11 (41)	27 (42)	15 (27)
Fatigue	9 (33)	21 (33)	23 (41)
Anemia	9 (33)	20 (31)	13 (23)
Nausea	8 (30)	13 (20)	14 (25)
Injection site pain	14 (52)	11 (17)	9 (16)
Chills	7 (26)	16 (25)	8 (14)
Decreased appetite	5 (19)	8 (13)	13 (23)
Constipation	3 (11)	13 (20)	8 (14)
Diarrhea	5 (19)	11 (17)	8 (14)
Vomiting	6 (22)	7 (11)	9 (16)
Dyspnea	4 (15)	10 (16)	7 (13)
Injection site reaction	2 (7)	8 (13)	9 (16)
Participants with ≥1 treatment-related AE	21 (78)	45 (70)	43 (77)
Most common treatment-related AEs^b^			
Pyrexia	9 (33)	21 (33)	11 (20)
Injection site pain	14 (52)	11 (17)	9 (16)
Chills	7 (26)	13 (20)	6 (11)
Fatigue	5 (19)	10 (16)	12 (21)
Injection site reaction	2 (7)	8 (13)	9 (16)
Participants with ≥1 immune-mediated AE	3 (11)	12 (19)	6 (11)
Infusion reactions	1 (4)	6 (9)	2 (4)
Hypothyroidism	2 (7)	2 (3)	2 (4)
Colitis	0	2 (3)	1 (2)
Severe skin reactions	0	3 (5)	0
Pneumonitis	0	1 (2)	1 (2)
Hyperthyroidism	0	1 (2)	0

All data are *n* (%) of participants. The safety population included all participants who received ≥1 dose of study treatment.

a Includes all AEs experienced while receiving IT MK-2118 + pembrolizumab in the 55 participants originally assigned to arm 2 and in the nine participants in arm 1 after crossing over to arm 2.

b Occurring in ≥15% of participants in any arm.

In arm 2 [*n* = 63, DLT-evaluable population (i.e., all participants who received ≥1 dose of treatment and were followed for ≥21 days or who had experienced a DLT)], a total of three DLTs (two grade 3 injection site reactions, one grade 3 cytokine release syndrome) were reported with the combination of IT MK-2118 plus IV pembrolizumab (Table 2). Similar to arm 1, there was no clear dose relationship. Sixty-two of the 64 participants (97%) treated with IT MK-2118 plus pembrolizumab in the safety population experienced AEs (Table 3). The most common AEs (>30% of participants) were pyrexia (42%), fatigue (33%), and anemia (31%). Treatment-related AEs were reported in 45 participants (70%) and were grade 3 or 4 in 15 participants (23%); there were no grade 5 treatment-related AEs. The only treatment-related AE that occurred in ≥25% of participants was pyrexia (33%).

#### Subcutaneous MK-2118 plus pembrolizumab (arm 4)

In arm 4 [*n* = 52, DLT-evaluable population (i.e., all participants who received ≥1 dose of treatment and were followed for ≥35 days or who had experienced a DLT)], one DLT (grade 3 pneumonitis) was reported with the combination of SC MK-2118 plus IV pembrolizumab at a relatively low dose of 20,000 μg MK-2118 (Table 2). All 56 participants treated with SC MK-2118 plus pembrolizumab in the safety population experienced AEs (Table 3). The only AE that occurred in >30% of participants was fatigue (41%). Treatment-related AEs were reported in 43 participants (77%) and were grade 3 or 4 in six participants (11%); there were no grade 5 treatment-related AEs. No treatment-related AEs occurred in ≥25% of participants.

### Pharmacokinetics

**Figure 1.**
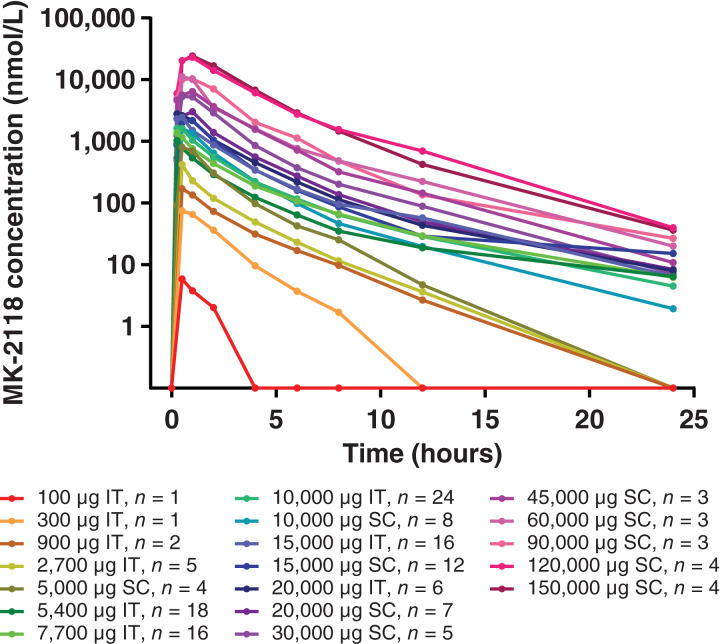
Arithmetic mean plasma concentration–time profiles of MK-2118 during cycle 1 following either IT (monotherapy and combination therapy with IV pembrolizumab) or SC (combination therapy with IV pembrolizumab) administration. MK-2118 concentration–time profiles for monotherapy and combination therapy arms were combined because there was substantial overlap in concentrations between arms.

Dose-dependent increases in systemic exposure of MK-2118 were observed following IT and SC administration (Fig. 1). The median systemic half-life of MK-2118 was approximately 3 hours (range, 1.6–3.9 hours) based on noncompartmental analyses of the plasma concentration–time profiles for MK-2118 following either IT administration, alone or with IV pembrolizumab, or SC administration with IV pembrolizumab.

### Efficacy

#### IT MK-2118 with or without IV pembrolizumab (arms 1 and 2)

**Figure 2.**
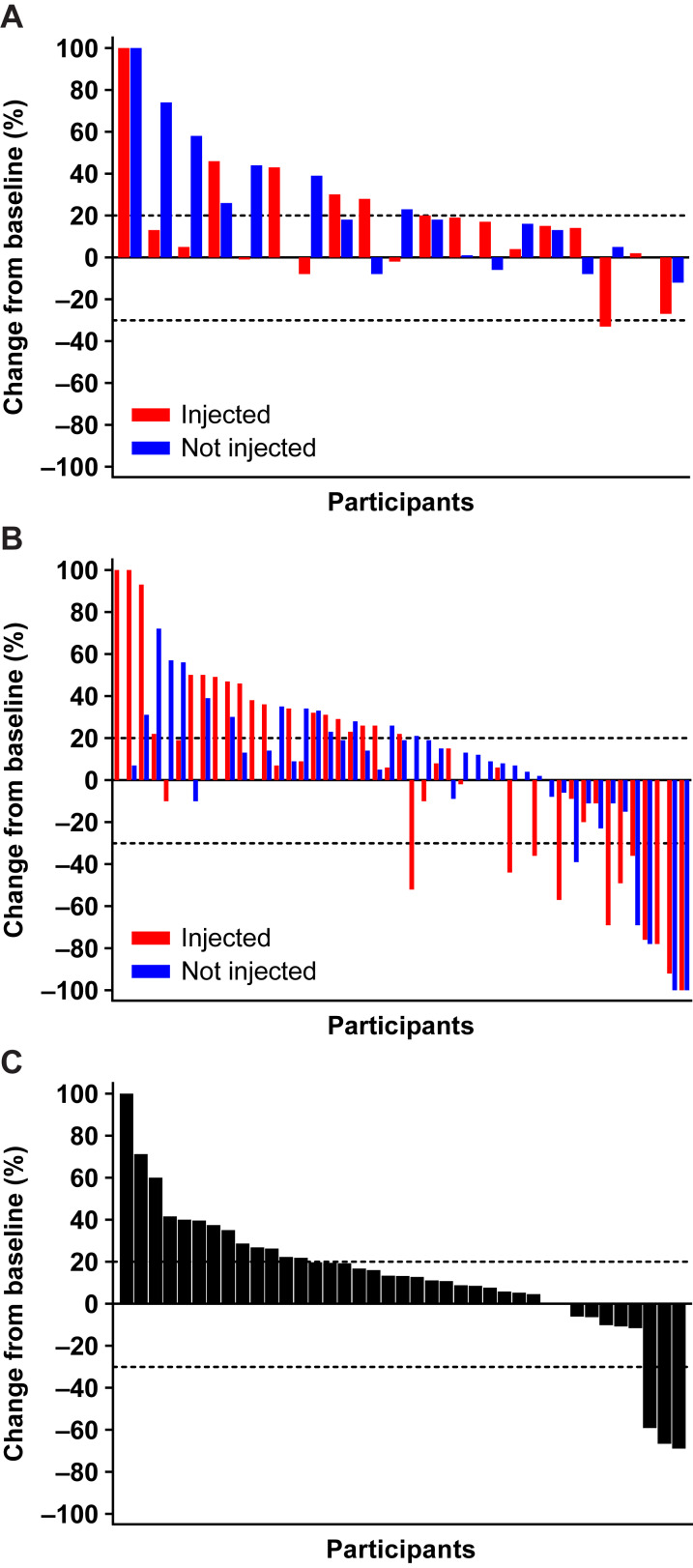
Best percentage change from baseline in target lesions per RECIST version 1.1 in (**A**) arm 1 (IT MK-2118 monotherapy), (**B**) arm 2 (IT MK-2118 plus IV pembrolizumab), and (**C**) arm 4 (SC MK-2118 plus IV pembrolizumab).

In arm 1 [*n* = 27, full analysis set population (i.e., all participants who received ≥1 dose of study treatment and had a baseline imaging assessment)], four participants (15%) had stable disease (SD) as the best response, 17 (63%) had PD, and 6 (22%) had no postbaseline assessment. Most participants had an increase from baseline in target tumor size in both injected and noninjected lesions (Fig. 2A; a ≥30% reduction was observed in the injected lesion of one participant). Median PFS was 2.0 months (95% CI, 1.6–2.5 months), and median OS was 4.9 months (95% CI, 3.4–8.3 months). The 6-month PFS and OS rates were 14% (95% CI, 4%–31%) and 40% (95% CI, 19%–62%), respectively.

In arm 2 [*n* = 63, full analysis set population (i.e., all participants who received ≥1 dose of study treatment and had a baseline imaging assessment)], ORR per RECIST version 1.1 was 6% (95% CI, 2%–16%), with one complete response (CR; participant with melanoma) and three partial responses (one participant each with melanoma, mesothelioma, and renal cell carcinoma). The participant with CR had received two prior lines of therapy (nivolumab/ipilimumab and then trametinib/dabrafenib) and had stage IV disease at baseline. Fourteen participants (22%) had SD, 33 (52%) had PD, 10 (16%) had no postbaseline assessment, and 2 (3%) had insufficient data for assessment of response. A ≥30% reduction from baseline in target tumor size was seen in the injected lesions of 11 participants and the noninjected lesions of five participants (Fig. 2B). Median PFS was 2.0 months (95% CI, 1.7–3.2 months), and median OS was 9.7 months (95% CI, 6.3–14.9 months). The 6-month PFS and OS rates were 23% (95% CI, 13%–36%) and 66% (95% CI, 51%–77%), respectively.

#### Subcutaneous MK-2118 plus pembrolizumab (arm 4)

In arm 4 [*n* = 53, full analysis set population (i.e., all participants who received ≥1 dose of study treatment and had a baseline imaging assessment)], ORR per RECIST version 1.1 was 3.8% (95% CI, 0.5%–13.0%), with one CR (participant with squamous cell carcinoma) and one partial response (participant with metastatic acinic cell carcinoma to the lungs). The participant with CR had received two prior lines of therapy (pembrolizumab and then pembrolizumab plus carboplatin/paclitaxel) and had stage IVA disease at baseline. Twelve participants (23%) had SD, 32 (60%) had PD, and seven (13%) had no postbaseline assessment. Most participants had an increase from baseline in target tumor size; a ≥30% reduction was seen for three participants (Fig. 2C). Median PFS was 1.9 months (95% CI, 1.8–2.1 months), and median OS was 9.4 months (95% CI, 6.8–12.0 months). The 6-month PFS and OS rates were 19% (95% CI, 10%–30%) and 68% (95% CI, 54%–79%), respectively.

#### Cutaneous T-cell lymphoma subgroup

Four participants in the study had CTCL and were included in the CTCL subgroup. Within this subgroup, two CRs (one each from arm 2 and arm 4) and two PDs (both in arm 4) were observed. The participant with CR in arm 2 had received eight prior lines of therapy (mechlorethamine hydrochloride, vorinostat, mogamulizumab, bexarotene, pralatrexate, bexarotene again, diphtheria toxin fragment IL2 fusion protein, and then imiquimod/bexarotene) and had stage II disease at baseline. The participant with CR in arm 4 had received one prior line of therapy (diphtheria toxin fragment IL2 fusion protein); baseline disease stage was not reported for this participant. The duration of response for the two participants who achieved a CR was 23.0 and 22.1 months, respectively, with both participants still responding at the time of treatment discontinuation.

### Biomarkers

**Figure 3.**
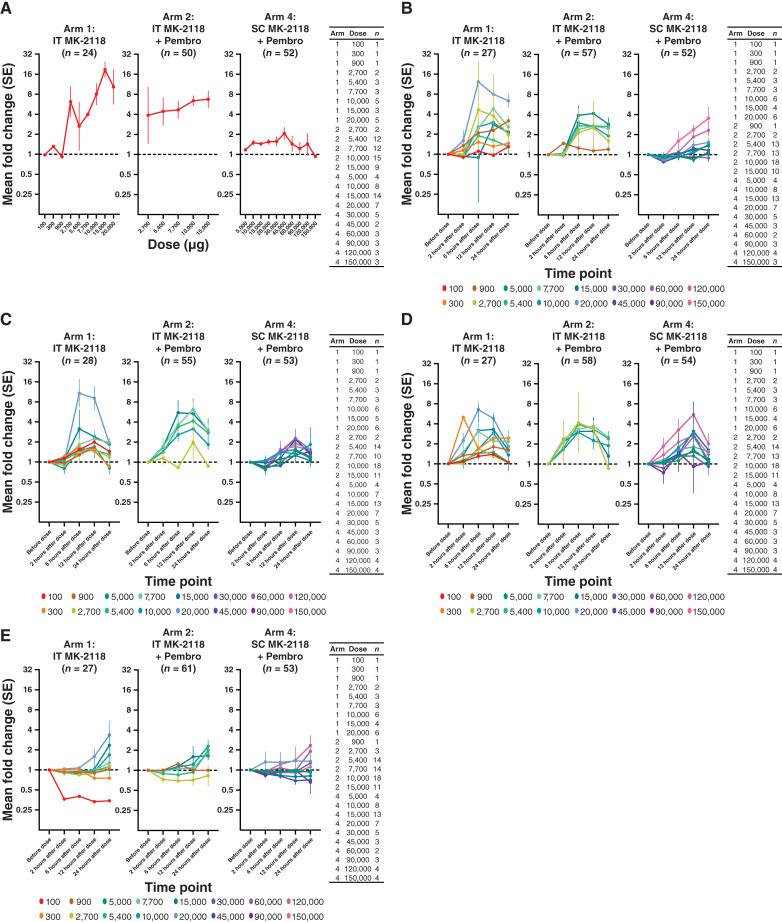
Geometric mean fold changes in (**A**) blood RNA, (**B**) IP-10, (**C**) IFNγ, (**D**) IL6, and (**E**) CRP in participants in arms 1, 2, and 4. Data are 6 hours postdose vs. predose for blood RNA and time point vs. predose for other biomarkers. pembro, pembrolizumab.

Following administration of IT MK-2118 in arms 1 and 2, dose-dependent changes were observed in STING-based blood RNA expression levels, IP-10, IFNγ, and IL6 (Fig. 3). No meaningful dose-dependent changes were observed in CRP. Following administration of SC MK-2118 in arm 4 (before pembrolizumab administration), dose-dependent changes were observed in IP-10 and IL6, but no meaningful dose-dependent changes were observed in STING-based blood RNA expression levels, IFNγ, or CRP (Fig. 3). In all arms, minimal changes in plasma concentrations were observed for IL1β, IL2, IL4, IL8, IL10, IL12p70, and IL13.

For the PD-L1 tumor proportion score, Spearman correlations with the best percentage change from baseline in target tumor size were 0.24 in arm 1, −0.27 in arm 2, and −0.30 in arm 4 (Supplementary Fig. S2A). For the PD-L1 combined positive score, Spearman correlations were not available for arm 1 (no samples), −0.52 in arm 2, and −0.23 in arm 4 (Supplementary Fig. S2B).

### Composite evaluation

Based on a composite evaluation of safety, pharmacokinetics, antitumor activity, and biomarkers, the maximum administered dose (MAD) of IT MK-2118 monotherapy (arm 1) was 20,000 μg, the MAD of IT MK-2118 administered in combination with IV pembrolizumab (arm 2) was 15,000 μg, and the MAD of SC MK-2118 administered in combination with IV pembrolizumab (arm 4) was 150,000 μg.

## Discussion

In this open-label, multicenter, multicohort dose escalation phase I study, repeated IT injection and systemic SC injection of MK-2118, a noncyclic dinucleotide STING agonist, with or without pembrolizumab, demonstrated feasibility with a manageable safety profile in participants with advanced solid tumors or lymphomas. Confirmation of STING pathway engagement, assessed by toxicity and pharmacodynamic testing, was well demonstrated with IT administration but unclear with systemic delivery despite comparable exposure for both IT and SC MK-2118. An interesting efficacy signal was noted in participants with CTCL, with two of the four participants achieving durable CRs.

Rapid and highly specific STING activation is consistently reported to amplify antitumor immunity through myeloid and dendritic cells but to also be dependent on adaptive immunity (particularly expansion of antigen-specific CD8^+^ T cells; refs. 1, 2). The priming and expansion of these CD8^+^ T cells via STING requires ongoing support and dependence on conventional type 1 dendritic cells (9–11). Murine studies have suggested a bell-shaped distribution of antitumor response versus deleterious effect on CD8^+^ T cells with some STING agonists, leading some to question whether higher exposures to STING agonists may dampen the generation of antigen-specific T cells (5). In this study, the impact of dose was less clear but rather we observed that route of administration seemed to be consequential and that even at systemically administered doses 10 times that of IT, no biologically active dose could be achieved. This was in contrast with preclinical modeling in which toxicity and lethality in animal models were achieved at doses that had no measurable effect upon systemic administration in humans. Higher doses of MK-2118 resulted in higher levels of peripheral blood type I IFN signature with IT but not SC administration despite a 10-fold difference in dose (Fig. 3A). This highlights the importance of local activation of STING in the tumor microenvironment. In contrast, systemic IP-10, IFNγ, and IL6 seemed to be dose proportional to systemic exposure with either modality. In totality, however, none of these pharmacodynamic changes were consistently associated with a clinical antitumor response. This raises potential questions surrounding the penetration of systemic delivery into the tumor microenvironment or perhaps feedback suppression mechanisms that limit the systemic expansion of type I IFN signaling.

Growing literature has identified both antitumor and protumor effects of STING (12). Chronic STING activation in tumor cells leads to chromosomal instability, yet it is also clear from preclinical models that STING agonists can drive local and systemic immunity (13). For example, the introduction of constitutively active mutant STING in *Mlh1*-knockout cells enhances type I IFN and adaptive immunity, supporting the antitumor properties of STING, particularly in DNA damage altered cancers (14). Moreover, although the systemic implications of local STING agonism are yet to be realized, local effects with associated translational support for driving type I IFN have been observed in clinical trials. A major open question remains surrounding the target cell for STING agonism. Some have argued that epigenetic silencing of STING in tumor cells limits their potential as clinical therapeutics, yet the preclinical literature has focused on myeloid and dendritic cell activation as being the primary targets for therapeutic effect (15).

Though our clinical data with MK-2118 are insufficient to support further development, a series of intravenously or systemically delivered STING agonists continue to proceed in clinical trials. It is notable that the development of the first-in-class IV molecule GSK-3745417, our subcutaneously administered molecule MK-2118, and the intramuscularly administered agent BMS-986301 are now discontinued, whereas no publicly available data have been disclosed for the IV STING agonist SB 11285. Interestingly, the IV STING agonist dazostinag continues in development having shown pharmacology and preliminary suggestion of antitumor activity (16). Looking forward, a series of novel approaches to deliver STING agonism are advancing. Examples include antibody–drug conjugates, notably targeting HER2 and C-C chemokine receptor type 2 (CCR2), lipid nanoparticles, and synthetic biology approaches via infectious vectors (salmonella; refs. 1, 17). It will be of interest to monitor the tumor cell-directed approach (HER2) as opposed to the myeloid-directed approaches (CCR2 and salmonella) to gain greater insight into the cell type of relevance for therapeutic STING targeting. Despite the observation that our systemically delivered noncyclic dinucleotide STING agonist had minimal impact, dose relationships with toxicity and potential benefit will also be important given a treatment-related death in the early experience of HER2-STING antibody-drug conjugate.

In summary, IT administration of MK-2118 triggered on-target effects of peripheral blood type I IFN but with limited clinical antitumor activity. SC administration did not demonstrate desired pharmacodynamic or clinical effects though data in participants with CTCL showed intriguing efficacy. These data will inform further development of STING agonists in cancer immunotherapy and help address open questions surrounding optimal cell type targeting and systemic feedback regulation of STING, selection of patients with cancer for therapeutic agonism, as well as the optimal route of administration.

## Supplementary Material

Supplementary Data 1 Table S1, Figure S1, Figure S2
