# Supplementary material for: Intratumoral or Subcutaneous MK-2118, a Noncyclic Dinucleotide STING Agonist, with or without Pembrolizumab, for Advanced or Metastatic Solid Tumors or Lymphomas
Source: Clin Cancer Res. 2025 Jan 23;31(7):1233–42. doi: 10.1158/1078-0432.CCR-24-2824 (PMC11964177; doi:10.1158/1078-0432.CCR-24-2824)

**Supplementary Table S1. Representativeness of Study Participants**

| Cancer type | Advanced or metastatic solid tumors or lymphomas |
| --- | --- |
| Considerations related to: | |
| Sex | In the US (2017–2021), incidence of cancer at any site was 478.7/100,000 males and 416.7/100,000 females |
| Age | In the US (2017–2021), cancer of any site was most frequently diagnosed in patients aged 65–74 years (30.2%), with a median age of 67 years at diagnosis |
| Race/ethnicity | In the US (2017–2021), incidence of cancer at any site was highest in non-Hispanic Black males (526.5/100,000) followed by non-Hispanic White males (510.7/100,000), non-Hispanic White females (447.0/100,000), non-Hispanic American Indian/Alaska Native males (442.2/100,000), non-Hispanic American Indian/Alaska Native females (421.1/100,000), and non-Hispanic Black females (403.1/100,000) |
| Geography | In the US (2021), incidence of new cancer cases was 427.8/100,000, with a mortality rate of 144.2/100,000; in Israel (2022), incidence of new cancer cases was 268.3/100,000, with a mortality rate of 83.2/100,000 |
| Other considerations | In the US (2024), estimates of new cases of cancer are 310,720 for breast cancer (female), 299,010 for prostate cancer, 234,580 for lung/bronchus cancer, 152,810 for colorectal cancer, and 100,640 for melanoma; estimates of deaths due to these cancers are 42,250; 35,250; 125,070; 53,010; and 8,290, respectively |
| Overall representativeness of this study | Patients in this study were primarily enrolled at US sites (1 site in Israel). The study population had a higher proportion of male (57%) than female patients, consistent with the incidence of cancer in the US. The median age (59 years) of study participants was younger than the median age at diagnosis in the general US population, but approximately one third were aged ≥65 years. Most study participants were White (78%), which is consistent with the US demographic (75% White). The most common tumor types in this study were colorectal cancer, breast cancer, and melanoma, which are among the most commonly diagnosed cancers in the US. Overall, the study population was generally representative of the real-world setting in locations where patients were enrolled |

**Supplementary Figure S1.** Patient disposition. ^a^Nine of the 27 patients in arm 1 crossed over to arm 2; these patients discontinued treatment due to progressive disease (n = 6), AEs (n = 2), and patient withdrawal (n = 1). AE, adverse event; IT, intratumoral; SC, subcutaneous.

**
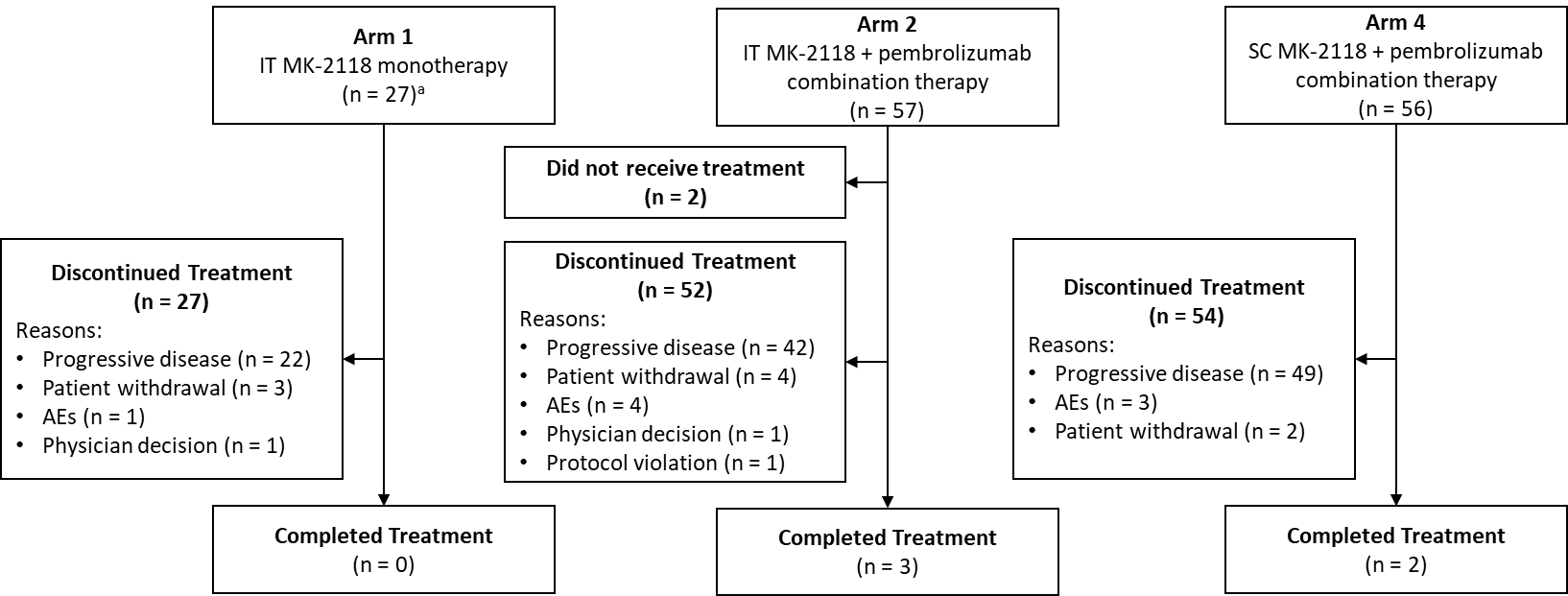
**

**Supplementary Figure S2.** Relationship between best percentage change from baseline in target tumor size per RECIST version 1.1 and (A) PD-L1 TPS and (B) PD-L1 CPS. Fitted lines (blue) and shaded regions (95% confidence intervals) were obtained via linear regression. CPS, combined positive score; IT, intratumoral; PD-L1, programmed cell death ligand 1; pembro, pembrolizumab; RECIST, Response Evaluation Criteria in Solid Tumors; SC, subcutaneous; TPS, tumor proportion score.


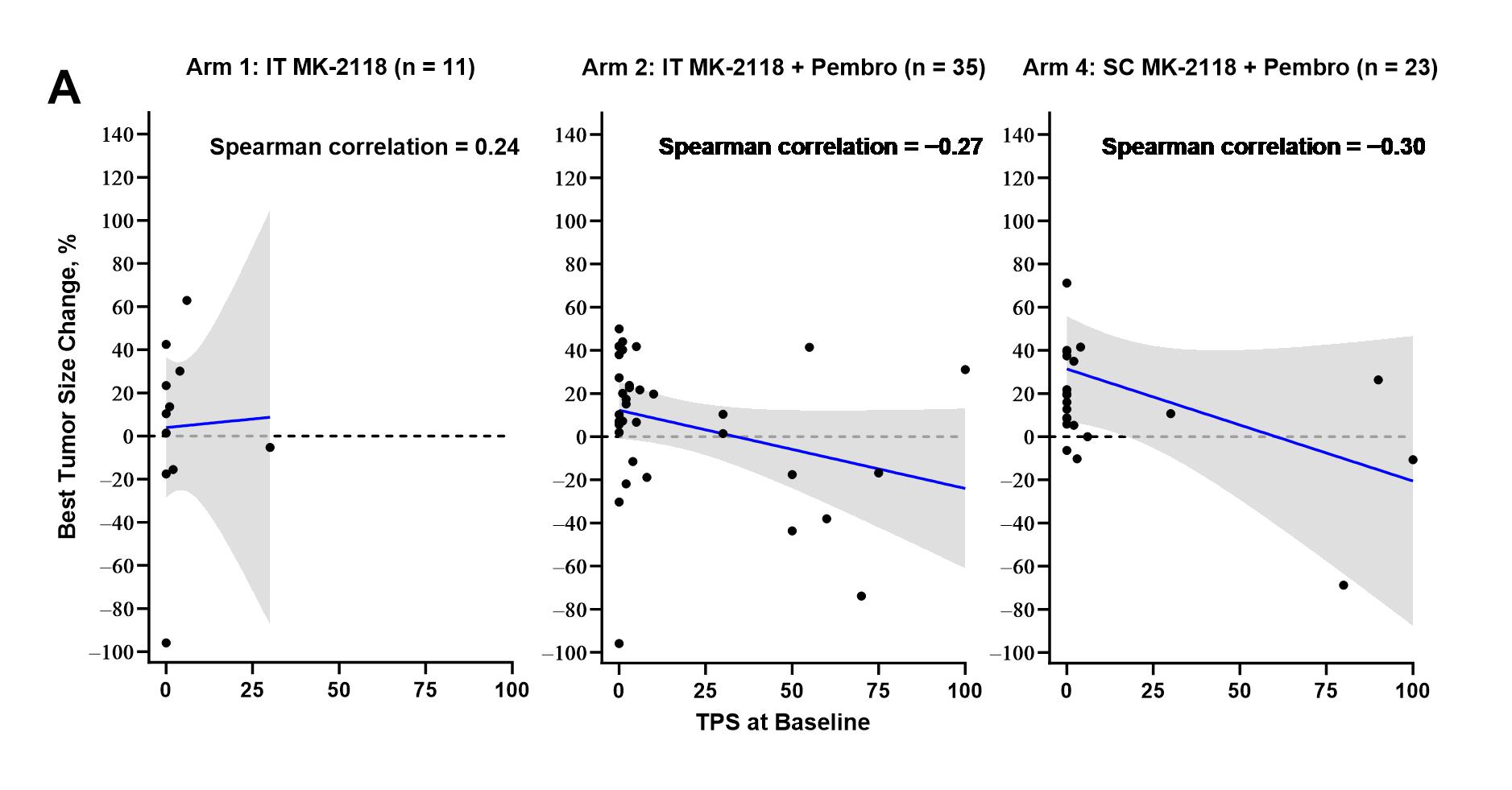


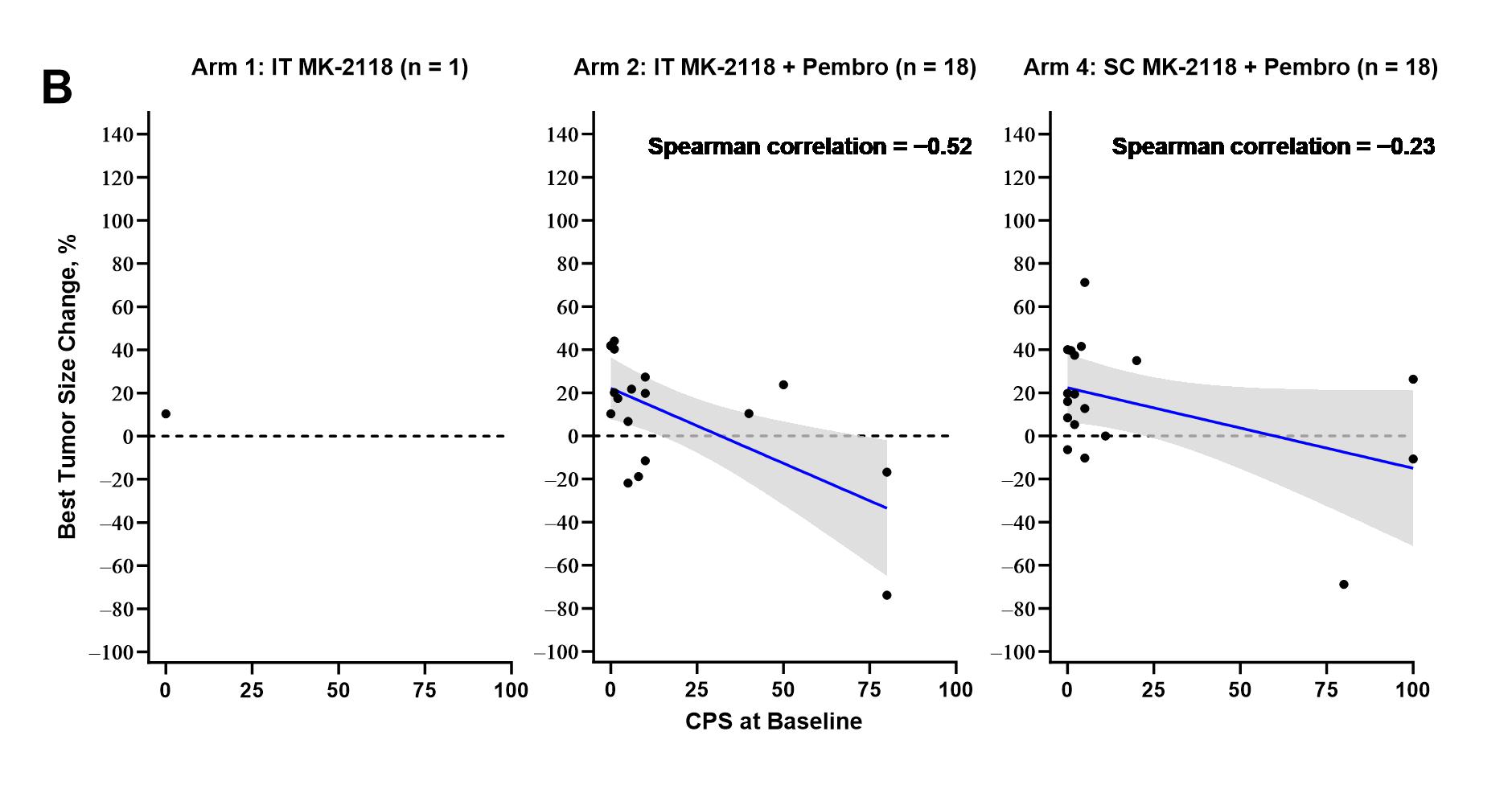

Supplement: Supplementary Data 1 — Table S1, Figure S1, Figure S2 [file ccr-24-2824_supplementary_data_1_suppds1.docx]
